# Decomposition of Secondary-Ion Kinetic Energy Distributions into Three Characteristic Components in MeV-SIMS

**DOI:** 10.1021/jasms.6c00280

**Published:** 2026-10-26

**Authors:** Boštjan Jenčič, Mitja Kelemen, Primož Pelicon

**Affiliations:** Jožef Stefan Institute, Jamova 39, 1000 Ljubljana, Slovenia

## Abstract

Kinetic energy distributions (KEDs) of secondary ions encode information about ion formation in secondary-ion mass spectrometry (SIMS), yet are typically interpreted on a per-ion basis without a unifying framework. In the MeV-SIMS regime, where sputtering is governed by electronic excitation within a transient ion track, the diversity of KED shapes reflects a complex and heterogeneous emission environment. Within this paper, we analyze an extensive data set of KEDs for atomic and molecular ions, including protonated, deprotonated, and cationized species of various organic molecules, using non-negative matrix factorization (NMF). We show how the data set of measured samples can be well described by three characteristic components. Dimensionality analysis demonstrates that three components provide the minimal description of the data, and the result is robust with respect to two different reconstructions of KEDs from yield–reflectron-voltage measurements. Mapping ion-specific weights in this three-component space can reveal systematic trends in KED fingerprints. Protonated ions are confined to the positive-energy regimes, whereas deprotonated ions exhibit a significant contribution from the negative-energy component, indicating additional reorganization processes. Atomic ions display a mass-dependent redistribution between the two positive-energy regimes, while the KED of cationized molecular ions is well aligned with the KED of the corresponding cation, especially for alkali metals. The three-component representation, therefore, provides a unified description of secondary-ion KEDs and is most likely consistent with emission under varying excitation conditions within the ion track. These results establish a direct link between kinetic energy distributions and ion formation pathways, offering a general framework for interpreting SIMS data across different ion classes and molecular systems.

## Graphical abstract

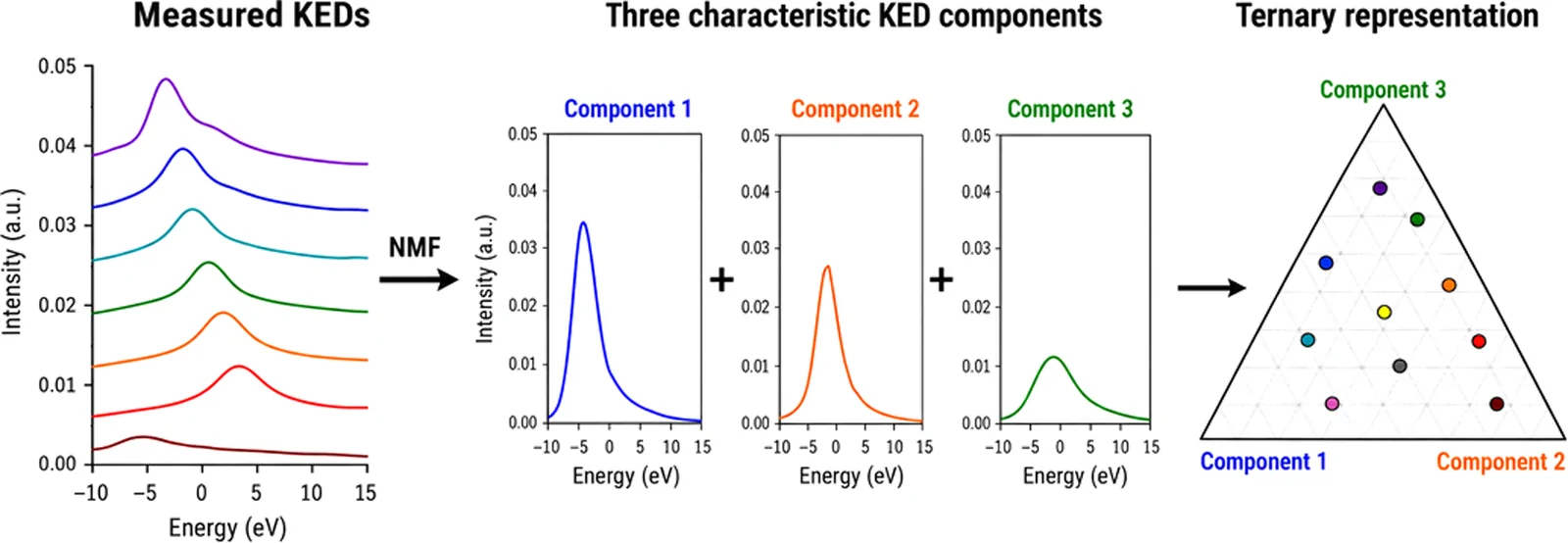

## 1 Introduction

Secondary-ion mass spectrometry (SIMS)^1^ is a widely used mass spectrometry imaging^2^ technique for surface chemical analysis, providing high sensitivity and enabling detection of both atomic and molecular species. It relies on the emission of atoms and molecules from the target due to the impact of the primary ion, which is a phenomenon known as sputtering. Since the onset of the method, various types of primary ions have been used in SIMS, based on its application scope, from originally used monatomic ions,^3^­[3] to polyatomic and cluster ions,^4–6^ and also ions of higher energy in the MeV range, a method known as MeV-SIMS.^7–9^

In the MeV-SIMS regime, sputtering is dominated by electronic stopping,^10^ leading to the formation of a transient ion track characterized by intense electronic excitation and rapid transfer of electronic energy to the lattice.^11^ The resulting highly nonequilibrium environment evolves on ultrafast time scales and exhibits strong spatial and temporal gradients in excitation density, temperature, and pressure.^12^ These conditions enable efficient emission of intact molecular ions,^13,14^ and also introduce significant complexity into the ion formation process. Ion emission is generally understood to involve varying degrees of energy dissipation, chemical rearrangement, coordination, and cluster-mediated processes.^15,16^

The kinetic energy distributions (KEDs) of emitted secondary ions offer insight into the processes governing sputtering and ion formation.^17–19^ The shape of a KED reflects the interplay between energy deposition, desorption dynamics, and ionization pathways and has been shown to depend strongly on ion type, molecular structure, and experimental conditions.^20^ Despite this, KEDs are typically analyzed individually, and a general framework linking different distributions to common underlying mechanisms has not been established.

Consequently, the diversity of observed KED shapes likely reflects contributions from multiple ion formation regimes associated with different local excitation conditions rather than a single uniform mechanism.

In this work, we analyze an extensive data set of KEDs for atomic and molecular ions, including protonated, deprotonated, and cationized species of various organic molecules, using non-negative matrix factorization (NMF). The non-negativity constraint is particularly suitable for KED analysis, as experimentally observed distributions can be represented as additive combinations of physically positive contributions. This approach therefore decomposes the data set into a small number of non-negative basis distributions and corresponding weights for each ion. We show that the KED data set can be described by three characteristic phenomenological components associated with excitation conditions and degrees of structural and chemical reorganization. The robustness of this decomposition is supported by dimensionality analysis and by independent reconstruction of KEDs from yield–reflectron-voltage measurements.

By mapping ion-specific weights within this three-component space, we reveal systematic trends that depend on the ion type, cationization mechanism, and molecular structure. The results demonstrate that complex KED behavior can be reduced to a small number of underlying emission regimes, providing a unified low-dimensional framework for describing the secondary-ion emission behavior in MeV-SIMS.

## 2 Methods

### 2.1 Sample Preparation

All samples were purchased from Sigma-Aldrich. Samples were prepared either as water solutions (amino acids, leu-enkephalin, epinephrine, poly­(ethylene glycol) (PEG)) or ethanol solutions (cholesterol) of concentrations between 1 and 5 mg/mL. Some arginine and PEG samples were also mixed with X-trifluoroacetate (X-TFA, X = Li, Na, K, and Ag) with a v/v ratio of 1, in order to test the effect of the adduct. Droplets of solution were deposited on a polished 1 × 1 cm^2^ Si wafer and spin-coated for 20 s at 2000 rpm. While the film thickness was not measured directly for the samples used in this study, previous measurements under comparable preparation conditions indicate film thicknesses exceeding 100 nm. Under such conditions, no measurable contributions from the Si substrate were observed in the mass spectra, and no systematic influence of film thickness on the measured KED can be detected.

### 2.2 MeV-SIMS Analysis

All analysis was done at the 2 MV tandem accelerator at the microanalytical center of Jožef Stefan Institute, Ljubljana, Slovenia. Due to the compromise between the high brightness, high electronic stopping contribution, and high focusing ability, we have employed 5 MeV ^35^Cl^5+^ primary ions for all measurements. Negative chlorine ions were accelerated into the tandem accelerator and stripped to the 5+ charge state, yielding a final primary ion energy of 5 MeV. While the energy and/or the electronic stopping power of the primary ion beam is another interesting parameter, which could be studied within this work, previous research^21^ exhibited a very weak impact of primary ion energy on KED within the possible range with the existing setup; therefore, we limited this study to only one primary ion beam type.

The resolution of the primary ion beam was approximately 15 × 20 μm^2^, which was achieved through closing the slit apertures within the beamline and finally by focusing the beam with triplet quadrupole magnetic lenses. The beam dimensions were estimated on a 200 periods per inch copper grid positioned in front of a channel electron multiplier (CEM) detector.

The Time-of-Flight mass spectrometry (Figure 1) was done through pulsing the primary ion beam to approximately 5 ns pulses every 100 μs. The introduction of pulsing reduced the primary ion current from 200 to 300 pA (measured by the charge integrator) to approximately 3000–5000 primary ions/s (measured by CEM), which is within the fA range.

**Figure 1:**
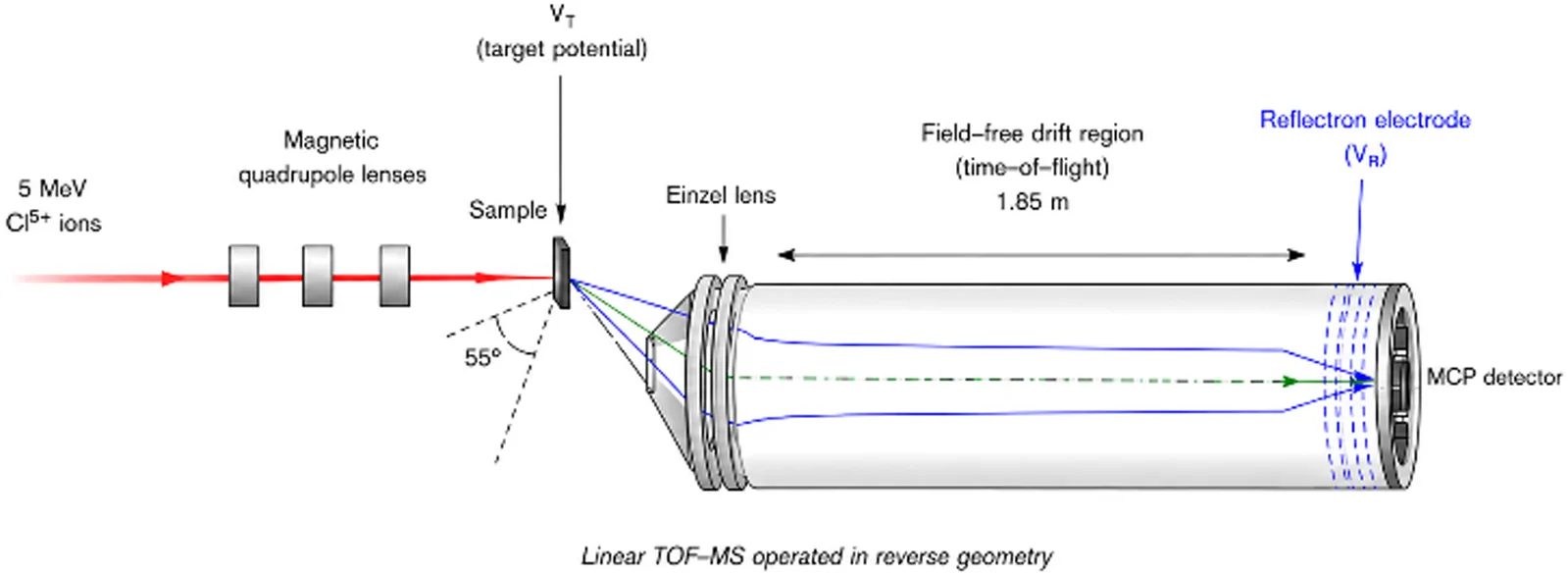
Scheme of the experimental setup for the MeV-SIMS analysis.

### 2.3 KED Measurement

After desorption, secondary ions were accelerated with a target voltage *V*_T_ of 3 kV into a time-of-flight mass spectrometer, which can be used either with a linear stage or a reflectron stage. Voltage on the reflection electrode *V*_R_ was used for the measurement of the KED, whereas spectra were monitored on the linear side. Due to the slight tilt of the reflection electrode, a careful calibration was needed in order to determine neutral voltage *V*_c_, a voltage where an ion with an energy of precisely e_0_ *V*_T_ would completely stop. The calibration was done by measuring the KED of H^+^ and H^–^ ions for target voltages between +1 and +5 kV (step of 1 kV) for positive voltages and between −3 and −5 kV (step of 500 V) for negative voltages. The measured slope of the linear function *V*_c_(*V*_T_) was 1.003 for both polarities, resulting in *V*_c_ = 3009 V for *V*_T_ = +3000 V and *V*_c_ = −4012 V for *V*_T_ = −4000 V, the target voltages used for the two polarities (also commonly used with the existing setup).

Each measurement of KED was done by scanning *V*_R_ from *V*_C_ – 20 V to *V*_c_ + 20 V, which reached both limits for most samples—total secondary-ion yield, and background only. Each measurement lasted 3–5 min, depending on the signal intensity of each analyte.

The scan size was approximately 500 × 300 μm^2^ (due to the rotation of the sample by 55 degrees, the *x*-axis scan was effectively larger). The combination of low primary ion currents and relatively large scan areas resulted in ion fluences of approximately 1.0 × 10^10^/cm^2^ h^–1^, well below the commonly accepted static SIMS limit (approximately 10^12^/cm^2^)^22^ for the duration of completing the whole set of measurements on one sample. Under these conditions, no measurable changes in signal intensity or spectral composition were observed upon repeated measurements, indicating negligible beam-induced damage or charging effects.

The intensity of the peaks was determined by integration after background subtraction. The spectral background was estimated by fitting an exponential decay function over the relevant mass range, excluding the ion peaks, and subsequently subtracted. From these data, we obtained the secondary-ion yield (*Y*) as a function of *V*_R_, which exhibits a monotonous decrease with increasing *V*_R_. Further analysis of KED was done in two separate ways: (a) numerical derivation of raw *Y*(*V*_R_) data, which resulted in increased noise due to the point-to-point derivatives when obtaining KED, and (b) fitting an empirical function to *Y*(*V*_R_) data and proceeding with analysis based on derivatives of fitted curves, which resulted in smaller errors. The details of the 3-parameter fitting curve can be seen in ref 21 The parallel analysis workflow was used to verify that the fitting procedure did not substantially influence the NMF composition.

Prior to NMF analysis, individual KEDs were normalized to unit integrated intensity and assembled into a matrix in which rows corresponded to individual ions and columns corresponded to kinetic energy channels. NMF decomposition was performed in Wolfram Mathematica using custom-written code based on non-negative least-squares minimization. Decompositions were evaluated for dimensions between one and ten components. The obtained basis distributions were found to be reproducible with respect to repeated runs and different subsets of ions, indicating that the extracted three-component structure represents a stable feature of the data set rather than a consequence of a particular initialization.

## 3 Results

The intensity evolution of a specific peak with varying V_R_ is shown in Figure 2 for the case of the protonated arginine molecular peak (*m*/*z* = 175). With *V*_R_ increasing from 0 V to approximately *V*_T_ – 10 V, the peak evolves through broadening due to the stronger effect of KED on the mass resolution. However, the integral below the peak remains the same. Afterward, the intensity decreases, and the rate of the intensity decrease is proportional to the KED, which is exhibited below.

**Figure 2:**
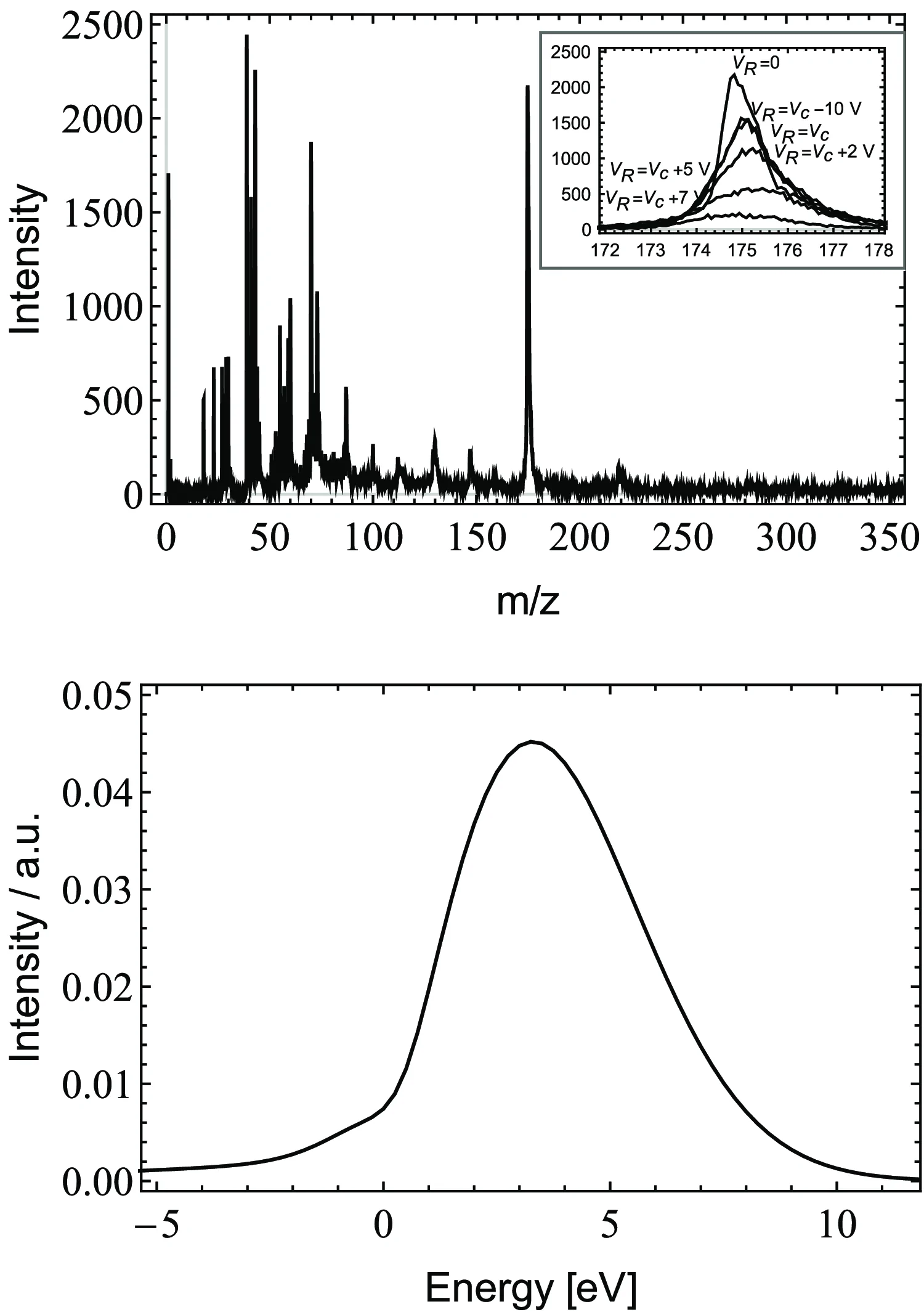
Above: Mass spectra of the arginine reference sample and the evolution of molecular peak intensity with increasing *V*_R_. The spectrum was recorded for 3 min, with approximately 5 × 10^5^ primary ions producing 3 × 10^4^ secondary ions of protonated arginine for *V*_R_ = 0 and *V*_R_ = *V*_c_ – 10 V (SIY = 5.6 × 10^–2^). Below: Fitted KED of the arginine molecular peak.

The arginine example illustrates the procedure used to obtain individual KEDs from yield–reflectron-voltage measurements. Applying the same workflow to all measured ions yielded a data set of 285 KEDs spanning from atomic ions to protonated and deprotonated molecular ions and various cationized species. The data set was subsequently analyzed using NMF to identify common underlying KED patterns. Because the data set is dominated by positive ions, the extracted basis distributions are primarily constrained by the positive-ion population; negative ions are included within the same basis to enable direct comparison of their KED characteristics. A separate NMF was also performed on the subset of 21 negative ions to evaluate the robustness of the common decomposition with respect to the ion polarity.

The dimensionality of the KED data set was evaluated using NMF with increasing rank both on raw KED data and on fitted KED data. As shown in Figure 3, the reconstruction quality improves rapidly when the number of components is increased from one to three, while further increases in dimensionality lead only to insignificant gains, which is especially evident for the fitted data. This behavior indicates that three components provide the lowest-dimensional description capable of capturing the essential structure of the data set, whereas higher-rank decompositions primarily refine peak shapes without introducing qualitatively new features.

**Figure 3:**
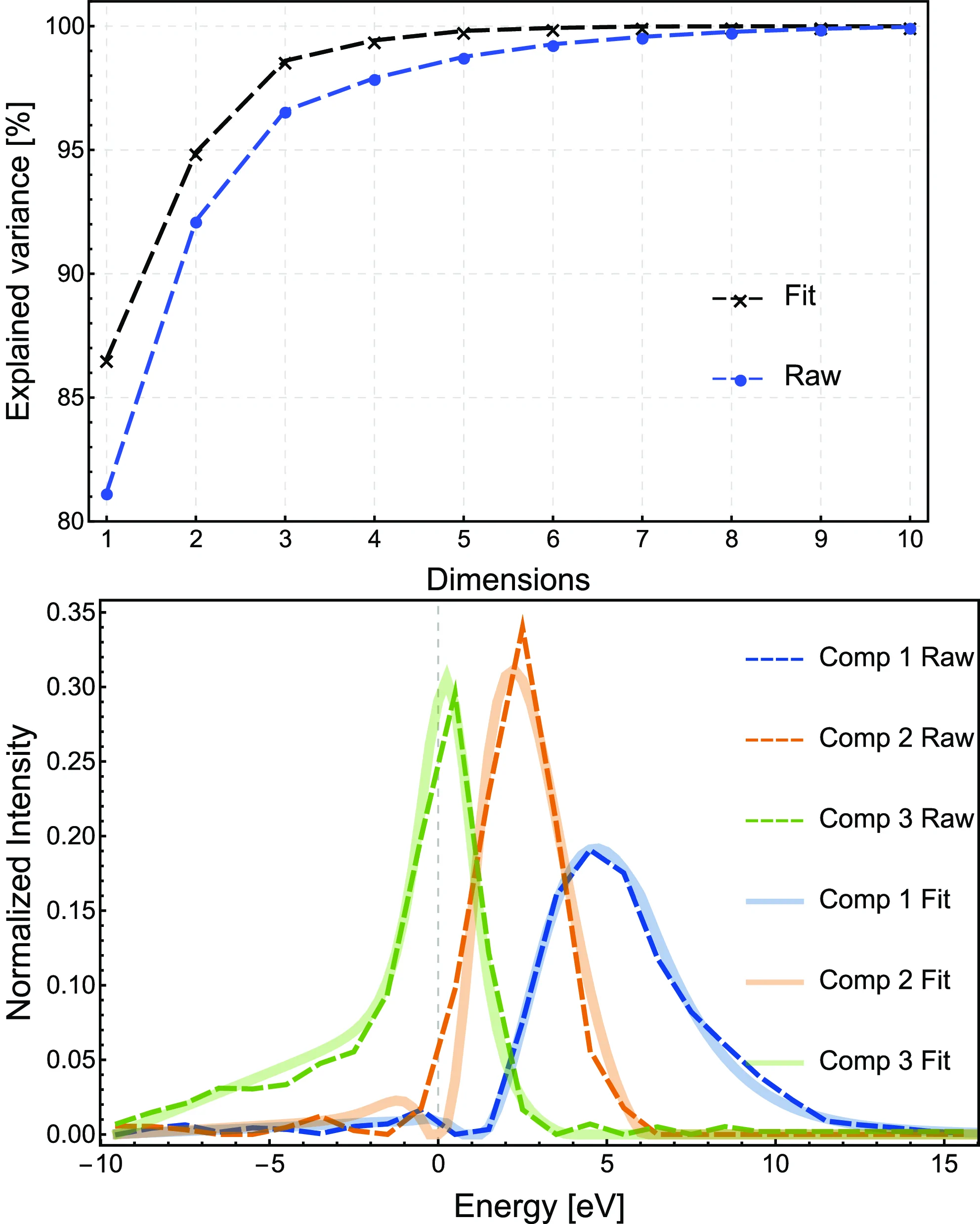
Above: Reconstruction quality as a function of the number of dimensions for raw KED data and fitted KED data. A rapid increase can be observed up to 3 dimensions. Below: The three base KED components in 3-dimensional space, raw and fitted KED data.

The three basis distributions obtained from the NMF analysis are also exhibited in Figure 3. Again, the types of distributions were obtained based on both raw data and fitted data. While the noise in individual raw KED shapes is significant in contrast to the fitted KEDs (therefore, the accuracy is consistently lower for all dimensions), the obtained basic distributions are very similar for both data sets. They can be broadly classified into:

(1) a high-energy component (Comp 1), characterized by a relatively broad distribution,
(2) a lower-energy component (Comp 2), narrower and shifted toward lower (but positive) kinetic energies,
(3) and a component with a pronounced negative tail (Comp 3).

These three components represent characteristic empirical basis distributions of secondary-ion KEDs for all measured samples and can also be reproduced through smaller sets of ions, e.g., through analysis of a single spectral peak only. A separate NMF of the 21 negative ions likewise yielded a qualitatively similar three-component solution, with only a marginal improvement in reconstruction quality compared to the common basis. The nature of base components in three dimensions can be further explained by the analysis of base components in two or four dimensions, which are shown in Figure 4.

**Figure 4:**
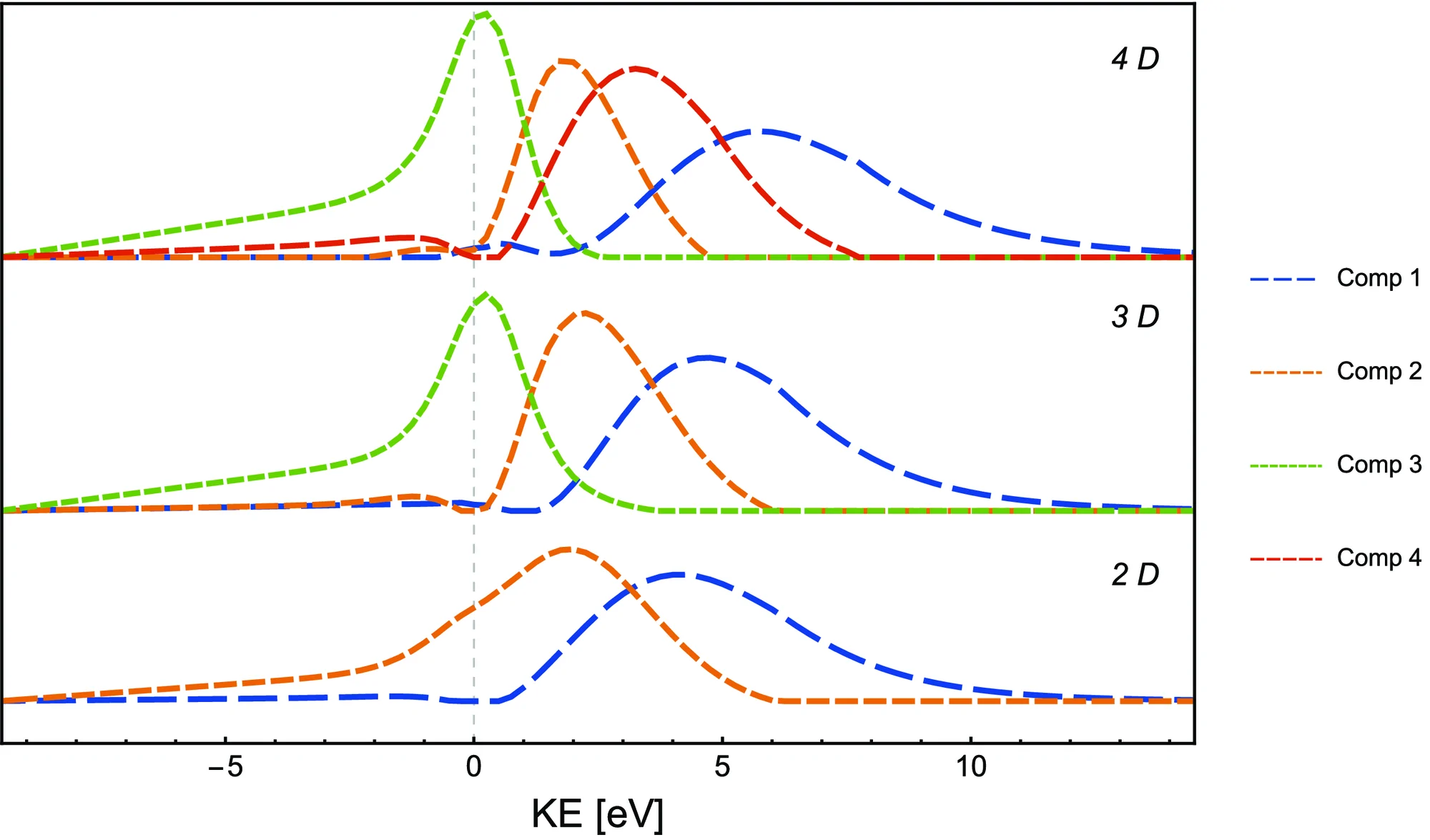
Base KED components in 2, 3, and 4 dimensions.

In two dimensions, the high-energy component remains conceptually the same, while the negative-tail component extends more into the positive region, seemingly adjusting for the missing Comp 2 in 3D space. While a negative tail contribution can be described reasonably well only with one base component, the variety of KEDs within the positive energy range cannot be sufficiently described only by Comp 1. Therefore, the third dimension yields the possibility to combine the contributions of high- and low-energy components (Comp 1 and Comp 2), which can be used to represent any given individual KED with much smaller errors.

Further, with an increase in the number of dimensions from three to four, the existing three components remain very similar. A newly added component, Comp 4, can be described as a medium-energy component but also has a mild negative tail. Its physical meaning is most likely not relevant, and its constitution is such that it improves only marginally the reconstruction accuracy of individual KEDs. In contrast, the three-component decomposition represents the minimal dimensionality that captures the essential structure of the data set while retaining phenomenologically meaningful basis distributions.

The observed trends suggest that Comp 1, Comp 2, and Comp 3 correspond to different secondary-ion emission regimes. Comp 1 and Comp 2 are both characterized by predominantly positive energy contributions and are therefore associated with comparatively less reorganized emission behavior. The difference between Comp 1 and Cop 2 is consistent with ion emission occurring under different excitation conditions within the evolving ion tracks. Within the framework of the thermal spike model, this behavior may reflect contributions from regions experiencing different excitation densities or different stages of track evolution. Comp 1 is therefore associated with comparatively higher energy emission conditions, whereas Comp 2 reflects comparatively lower energy emission behavior and/or increased energy redistribution prior to emission.

Importantly, neither Comp 1 nor Cop 2 alone is sufficient to describe the diversity of experimentally observed KEDs. Instead, most ions occupy intermediate positions between these two components, indicating that the measured KEDs arise from the continuous mixing of high- and low-energy emission regimes.

In contrast, Comp 3 is distinguished by a pronounced negative tail extending toward lower apparent kinetic energies, consistent with ions that do not fully experience the nominal extraction potential. Such distributions are difficult to explain solely by variations in direct emission energy and instead suggest the involvement of additional processes beyond simple desorption. Comp 3 is therefore consistent with ion formation pathways involving increased degrees of structural or chemical reorganization, clustering, charge transfer, or postemission complex evolution.

The physical interpretation of the extracted components should be regarded as phenomenological rather than being uniquely mechanistic. While the observed trends are consistent with ion emission occurring under different excitation and reorganization conditions within the evolving ion track, the present analysis does not directly resolve the spatial or temporal origins of the individual components. The extracted basis distributions, therefore, represent empirical characteristic KED signatures that provide a compact, low-dimensional framework for describing secondary-ion emission behavior. The utility of the three-component representation becomes particularly apparent when comparing different classes of secondary ions.

The ternary representation of protonated and deprotonated molecular ions is shown in Figure 5. A clear separation between positive and negative-ion species can be observed with respect to the contribution of comp 3. Protonated molecules are clustered predominantly along the edge connecting Comp 1 and Comp 2, indicating that their KEDs can be described almost entirely as mixtures of the two positive energy components. This behavior suggests that protonated ion formation is dominated by emission regimes associated with comparatively lower degrees of structural and chemical reorganization.

**Figure 5:**
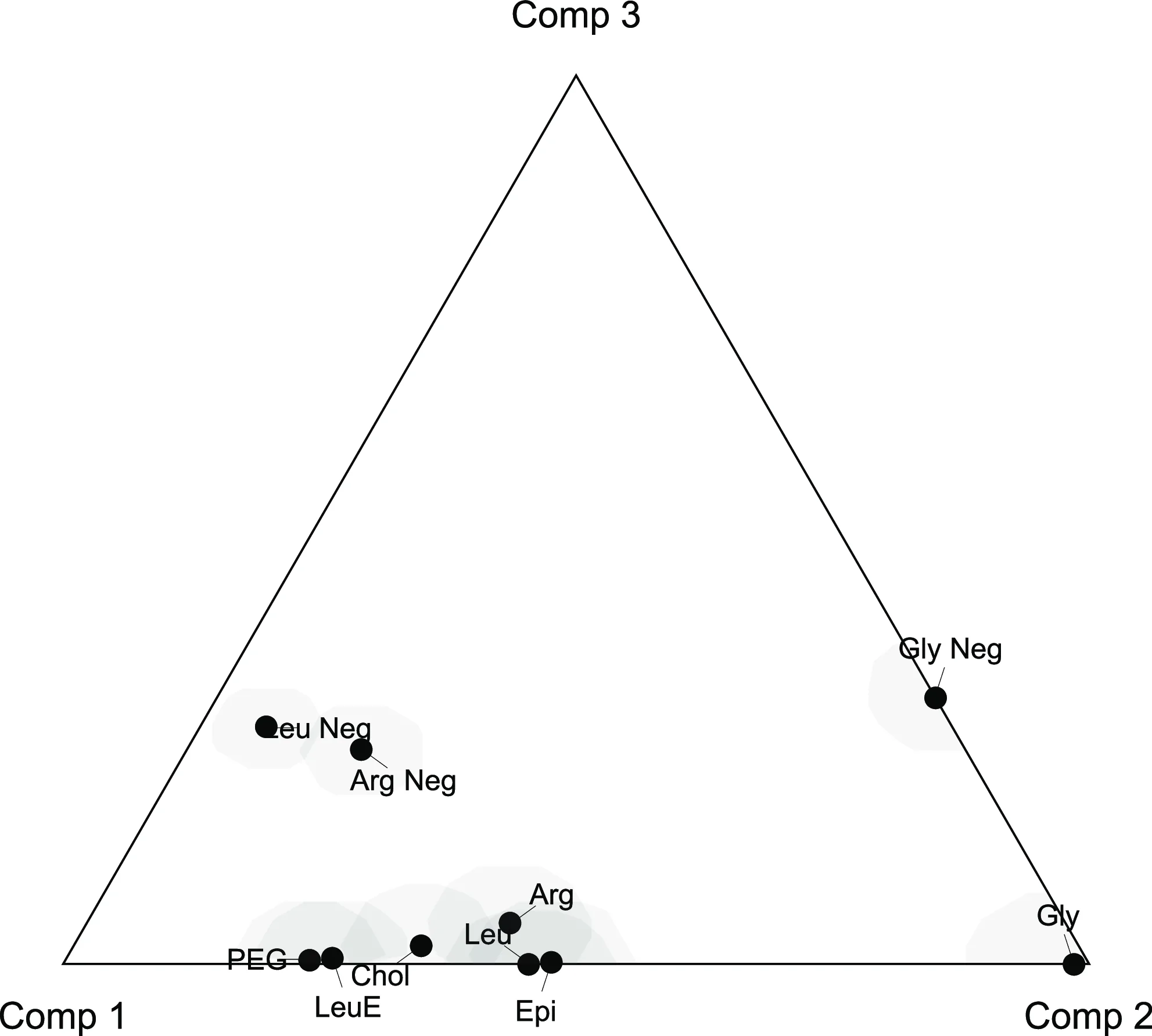
Ternary plot of various protonated (M + H, only labeled as “M”) and deprotonated (M – H, labeled “M neg”) organic molecules. A clear difference in the contribution of Comp 3 is visible between protonated and deprotonated species (positive vs negative mode).

In contrast, deprotonated ions exhibit a substantial Comp 3 contribution. Both arginine and leucine negative ions are shifted strongly toward the Comp 1–Comp 3 edge, while glycine negative ions lie almost entirely along the Comp 2–Comp 3 edge. This indicates that negative-ion formation involves significantly different emission conditions compared to protonated species. The noticeable contribution of Comp 3 may reflect the involvement of processes associated with increased structural or electronic reorganization, such as electron capture, delayed charge separation, or cluster-mediated ion formation.

The relatively broad estimated uncertainty regions indicate partial overlap between neighboring emission regimes, but the separation between the positive- and negative-ion populations remains robust. The results, therefore, suggest that Comp 3 corresponds to a distinct ion formation regime that is preferentially sampled during negative-ion formation, whereas protonated molecular ions are formed predominantly under conditions associated with the two positive-energy emission components.

The ternary representation of atomic ions and cationized molecular species is shown in Figure 6. The atomic ions form well-defined clusters that reveal systematic trends related to the ion type and mass. Hydrogen ions obtained from different molecular systems cluster tightly near the Comp 1 corner, demonstrating that H^+^ KEDs are highly reproducible and are only weakly dependent on the chemical environment from which they originate. This is consistent with the proton emission being governed predominantly by highly energetic emission conditions associated with comp 1.

**Figure 6:**
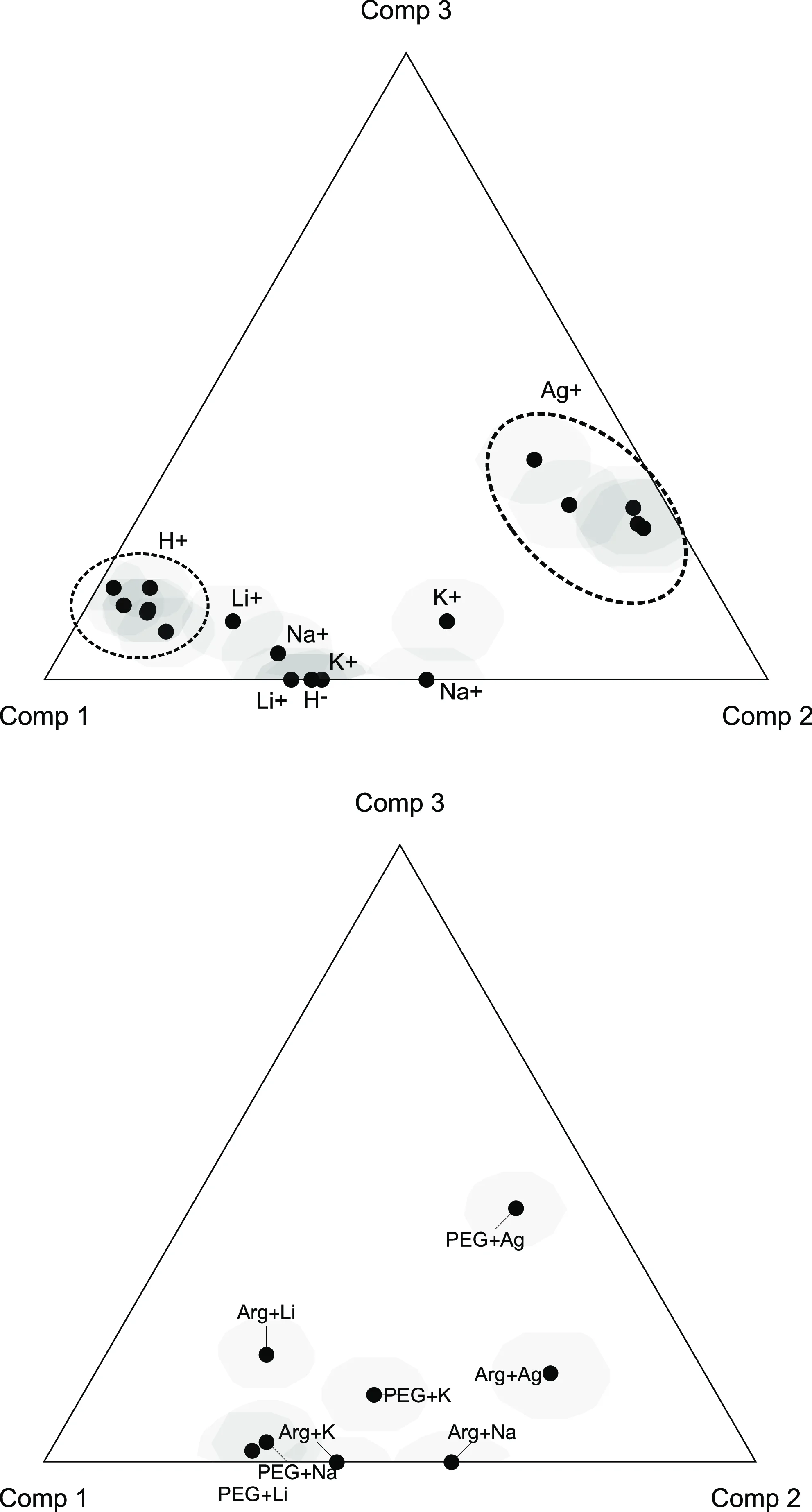
Ternary plot of various atomic ions from different samples (above) and for differently cationized PEG and arginine molecules (below).

In contrast, alkali ions exhibit progressively larger contributions from Comp 2 with increasing ion mass. Li^+^ remains relatively close to the H^+^ cluster, while Na^+^ and particularly K^+^ shift toward the Comp 2 corner. This trend indicates a redistribution from higher energy toward lower-energy positive KED components, consistent with increasing energy dissipation and stronger coupling of heavier ions to the surrounding medium prior to emission. Nevertheless, all alkali ions remain confined close to the Comp 1–Comp 2 edge, indicating negligible participation of the Comp 3 regime.

Silver ions behave distinctly differently from those of alkali species. Ag^+^ forms a separate cluster characterized by a substantial Comp 3 contribution. This behavior indicates that silver emission involves additional ion formation processes involving increased degrees of reorganization and energy redistribution. The pronounced Comp 3 contribution is consistent with the strong tendency of Ag to participate in coordination and cluster-mediated interactions during secondary-ion formation.^23,24^

The corresponding cationized molecular ions exhibit trends closely related to those observed for the isolated atomic ions, especially for the alkali metals. Li^+^-, Na^+^-, and K^+^-cationized species occupy regions similar to those of the corresponding atomic ions, while Ag-cationized species again exhibit increased contribution from Comp 3 compared to alkali-metal adducts. This close correspondence suggests that cationized molecular ions retain KED characteristics associated with the attached cations to a significant degree.

In contrast to protonated molecular ions, which are distributed predominantly along the Comp 1–Comp 2 edge independently of H^+^ itself, cationized species exhibit distributions more closely correlated with those of their associated atomic ions. At the same time, differences between PEG and arginine demonstrate that the molecular structure additionally modulates the relative contributions of the three emission regimes. The ternary representation, therefore, indicates that both the identity of the cation and the chemical properties of the molecular system influence the final KED shape.

The ternary representation of PEG-derived ions is shown in Figure 7. Two clearly separated and internally ordered populations can be observed, corresponding to the previously identified A (*M*_n_) and B (*M*_n_-H_2_O) ion series.^25^ Despite originating from the same molecular system, the two series occupy distinct regions within the three-component space, indicating a substantially different ion formation behavior.

**Figure 7:**
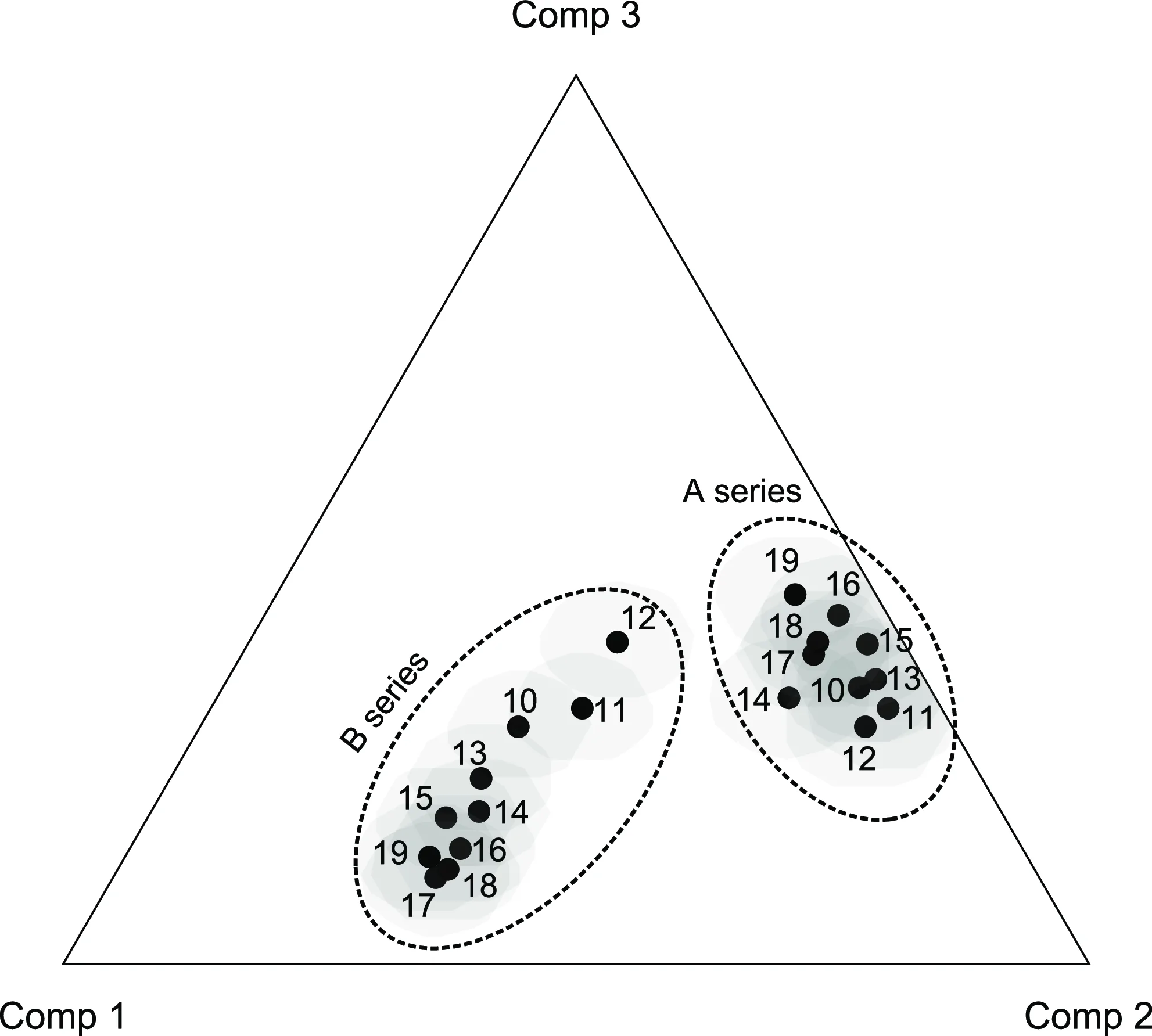
Ternary plot of PEG A- and B-series.

Both ion series exhibit substantial contributions from Comp 3, demonstrating that PEG ion formation is associated with emission regimes involving increased energy redistribution and/or molecular reorganization compared to most protonated molecular ions. However, the relative balance between the two positive-energy components differs significantly between the two PEG populations. The A series is shifted toward the Comp 2–Comp 3 side of the diagram, whereas the B-series exhibits a comparatively larger Comp 1 contribution and is displaced more toward the Comp 1–Comp 3 edge.

An important observation is that both PEG series form compact and approximately parallel trajectories in the ternary space. Within each population, the relative contributions of the three components evolve gradually with the oligomer size rather than changing discontinuously between neighboring ions. This behavior suggests that the observed KEDs are governed by continuous variations in energy redistribution and ion stabilization processes as the molecular mass increases.

The existence of two distinct yet internally ordered PEG populations demonstrates the sensitivity of the three-component representation to subtle differences in the ion formation behavior. Although the exact microscopic origin of the A- and B-series cannot be established unambiguously from the present data alone, the results indicate that the two ion families are associated with different balances of excitation, energy redistribution, and molecular reorganization during secondary-ion emission. The ordered arrangement of PEG oligomers further supports the interpretation that the extracted components represent physically meaningful emission trends, rather than mathematical decomposition artifacts.

## 4 Conclusion

In this work, a large data set of secondary-ion kinetic energy distributions obtained in the MeV-SIMS regime was analyzed using non-negative matrix factorization. The results demonstrate that the experimentally observed diversity of KED shapes can be represented using three characteristic phenomenological components, which together provide a compact and robust description of the secondary-ion emission behavior across a broad range of atomic and molecular ions.

Dimensionality analysis showed that three components represent the minimal decomposition capable of capturing the essential structure of the data set. Lower-dimensional decompositions failed to adequately reproduce the diversity of positive-energy KED shapes, while higher-dimensional solutions produced only marginal improvements and introduced additional components without clear phenomenological significance. The three-component representation was further shown to be robust with respect to the method of KED reconstruction from yield–reflectron-voltage measurements.

Mapping individual ions within the resulting ternary space revealed systematic trends related to the ion type, molecular structure, ion polarity, and cationization mechanism. Protonated molecular ions were found predominantly along mixtures of the two positive energy components, whereas deprotonated species exhibited substantial contributions from the component characterized by a pronounced negative tail. Atomic ions displayed systematic redistribution between the two positive-energy components with increasing ion mass, while Ag-containing species consistently showed an increased contribution from the negative-tail component. PEG ion series formed two distinct yet internally ordered populations, demonstrating the sensitivity of the approach to subtle differences in the ion emission behavior.

The observed trends are consistent with the interpretation that the three extracted components reflect different excitation and reorganization conditions during secondary-ion emission under electronic sputtering conditions. While the exact microscopic origin of the extracted components remains unresolved, the present results demonstrate that complex KED behavior can be represented within a unified, low-dimensional phenomenological framework.

The extracted KEDs should nevertheless be regarded as experimentally measured distributions that may contain contributions from the extraction geometry and ion-transmission characteristics of the instrument, rather than as strictly intrinsic distributions of the emitted secondary ions. However, the previously observed invariance of most KED shapes over a broad range of target potentials (1–4 kV)^21^ suggests that the principal KED characteristics identified here are not dominated by extraction-field-dependent transmission effects. Overall, the proposed approach establishes kinetic energy distribution decomposition as a useful tool for investigating secondary-ion emission behavior in SIMS. More broadly, the results demonstrate that complex secondary-ion KEDs exhibit an unexpectedly ordered low-dimensional structure, enabling systematic comparison of ion emission behavior across different molecular systems, ion types, and sputtering conditions. Research of additional parameters, such as primary ion energy, charge state, and species, can also be analyzed via such an approach in the future.
